# Crystallized and fluid cognitive abilities have different genetic associations with neuropsychiatric disorders

**DOI:** 10.21203/rs.3.rs-5256724/v1

**Published:** 2025-01-24

**Authors:** Diego Londono-Correa, Javier de la Fuente, Gail Davies, Simon Cox, Ian Deary, K Harden, Elliot Tucker-Drob

**Affiliations:** University of Texas at Austin; Department of Psychology, University of Texas at Austin; Department of Psychology, University of Edinburgh; University of Edinburgh; University of Edinburgh; University of Texas at Austin; The University of Texas at Austin

**Keywords:** Psychiatric disorders, cognitive function, intelligence, educational attainment, noncognitive skills, GWAS-by-subtraction

## Abstract

Cognitive function is associated with risk for multiple neuropsychiatric disorders. Previous research on the genetic relations between cognition and psychopathology has largely treated cognitive function as unitary, in part due to a lack of well-powered genome-wide association studies (GWAS) on specific domains, particularly crystallized knowledge (Gc). Important domains within the hierarchy of cognitive function, especially Gc, have been underexplored regarding their associations with psychiatric disorders. Here, we parse the genetics of cognitive test performance into components representing reaction time, fluid reasoning, and crystallized knowledge. This multivariate approach that allows us to report results from a GWAS meta-analysis of crystallized knowledge (N ~ 438,000). We then test how multiple neuropsychiatric disorders with established links to cognitive function (Schizophrenia, Bipolar Disorder, Autism Spectrum Disorder, Attention Deficit Hyperactivity Disorder, and Alzheimer’s Disease) are genetically related to these three cognitive domains, and to a noncognitive factor associated with educational attainment (NonCog). We document specific and heterogenous patterns of genetic associations between each neuropsychiatric disorder and the different domains of cognitive function and the noncognitive factor. Previous reports of genetic sharing between neuropsychiatric disorders and GWAS of aggregate cognitive function or educational attainment have failed identify these substantial differences in which cognitive functions drive these relations for which disorders.

## Introduction

Many neuropsychiatric disorders involve cognitive impairments that impede performance in education and other social roles ^1^. Cognitive impairments are also evident in asymptomatic individuals prior to the onset of neuropsychiatric disease, and among unaffected family members ^2–4^. Results from genome-wide association studies (GWASs) have found that cognition and psychopathology partially share a genetic basis. Multiple neuropsychiatric disorders, including attention deficit/hyperactivity disorder (ADHD), autism spectrum disorder (AUT), Alzheimer’s disease (ALZ), bipolar disorder (BIP) and schizophrenia (SCZ) show genetic correlations with performance on tests of general cognitive function^5,6^

Although previous genomic research on the relationship between cognitive function and neuropsychiatric disorders has largely treated cognitive function as a unitary trait, cognitive function can be differentiated into separable (though correlated) domains, including processing speed (measured by tests of reaction time, RT), fluid reasoning (Gf), and crystallized knowledge (Gc)^7^. RT is thought to be a relatively basic function representing the rapidity with which simple cognitive tasks are completed correctly; shorter reaction times are associated with better scores on tests of more complex cognitive processing^8–11^. Gf is a domain of higher-order cognitive function, defined as the ability to effortfully process complex information and to solve novel problems ^7^. Gc is another domain of higher-order cognitive function, and involves the relatively automatic retrieval or application of knowledge or information previously acquired through culture and experience ^12^. Both twin and genomic studies indicate that RT, Gf, and Gc have correlated but non-synonymous genetic architectures ^13–15^. They also show diverging age trends: RT and Gf exhibit monotonic mean declines throughout adulthood, whereas Gc exhibits mean gains through approximately the seventh decade of life ^16–20^.

Evidence from research outside of genomics consistently indicates that the cognitive impairments evident in neuropsychiatric disorders are best characterized at the level of specific domains of cognitive function, rather than as uniform and global deficits. For instance, patients with SCZ show more pronounced deficits on tests of fluid reasoning (Gf) than on tests of crystallized knowledge (Gc) ^21^. Work in genomics has reported pairwise genetic correlations between individual cognitive phenotypes and individual neuropsychiatric disorders, comparisons of which provide initial evidence that genetic associations may be heterogenous across different domains of cognitive function. Hagenaars et al.^22^ reported that SCZ, ALZ, and AUT were genetically associated with lower scores a verbal-numerical reasoning test (a hybrid test of crystallized knowledge and fluid reasoning) though only SCZ was also linked to slower reaction time. Similarly, Carey et al. ^23^ reported stronger negative genetic associations of schizophrenia with the digit symbol substitution test (a test of processing speed) than with a test of Vocabulary knowledge. De la Fuente et al. ^14^ reported that the negative genetic association between SCZ and general cognitive ability was weaker when tests of processing speed were excluded from the general ability factor, whereas the negative genetic association between ADHD and general cognitive ability was stronger when excluding tests of processing speed. Rajagopal et al. ^24^ decomposed school grades into math and language-specific dimensions, revealing that while polygenic scores for math grades showed negative associations with several psychiatric disorders, polygenic scores for language grades positively correlated with SCZ and BIP risk. This aligns with findings from educational attainment (EA) where evidence also points to heterogenous genetic influences across cognitive and noncognitive domains ^25^.

Several studies have observed positive genetic associations between EA and neuropsychiatric disorders, including SCZ and AUT, which contradicts the known functional impairments seen in patients with these disorders ^22,26–30^. One explanation for these paradoxical associations is that education is genetically heterogeneous, involving multiple domains of cognitive function, as well as “noncognitive” factors such as motivation, persistence, creativity, and openness^25^. Parsing the genetic heterogeneity of educational attainment in relation to neuropsychiatric disorders by way of multiple cognitive and noncognitive pathways has the potential to illuminate these apparently paradoxical results.

Previous studies, however, have been relatively narrow in the sets of cognitive variables examined, and have only estimated genetic associations between neuropsychiatric disorders and individual cognitive phenotypes using bivariate methods. One major challenge to testing the genetic relations between neuropsychiatric disorders and more specific domains of cognitive function has been a lack of well-powered GWAS data on individual domains, particularly crystallized knowledge. Here, we address this challenge by implementing a multivariate approach that decomposes genetic associations between multiple cognitive functions and educational attainment into independent variance components representing reaction time, fluid reasoning, crystallized knowledge, and a noncognitive factor related to higher education (which we label “NonCog”). Our methodology expands the GWAS-by-subtraction approach introduced by Demange et al. ^25^ within the Genomic Structural Equation Modeling (Genomic SEM^31^) framework to the multivariate setting, decomposing genetic associations among a set of GWAS phenotypes into multiple orthogonal components. The Genomic SEM framework allows us, simultaneously, to conduct a well-powered GWAS of crystallized knowledge. We then examine the genetic correlations between these cognitive domains, non-cognitive skills related to educational attainment, and five neuropsychiatric disorders known to be associated with cognitive decrements: Schizophrenia, Bipolar Disorder, Autism Spectrum Disorder, Attention Deficit Hyperactivity Disorder, and Alzheimer’s Disease.

## Results

### Curation of GWAS related to cognition, education, and neuropsychiatric disorders

We selected GWAS results that were relevant to RT, Gf and Gc cognitive abilities. This resulted in summary statistics for 10 cognitive phenotypes, ranging from reaction time (RT^14^), tests of Gf (matrix pattern recognition^14^, tower rearranging^14^, trail making test-B^14^), verbal numerical reasoning^14^ (which combines Gf and Gc), and tests of Gc (vocabulary synonyms^23^, word reading^32^, nonword reading^32^, phoneme awareness^32^, spelling^32^). We also conducted an additional GWAS of recently released data for picture vocabulary, a test of Gc, from the UK Biobank (see methods for details). See Table 1 for a list of all the phenotypes.

In addition, we obtained summary statistics for two educationally relevant personality traits, openness to experience^33^ and conscientiousness^33^, and for educational attainment.

Finally, we obtained GWAS summary statistics for five neuropsychiatric disorders with known associations with impaired cognitive function at the phenotypic and genetic levels (Schizophrenia ^34^, Bipolar disorder^35^, Autism spectrum disorder^36^, Attention deficit hyperactivity disorder^37^, Alzheimer’s disease^38^). All the GWAS summary statistics used in this study come from individuals of European Ancestry.

### Confirmatory genetic factor model of cognitive function

**Figure 1:**
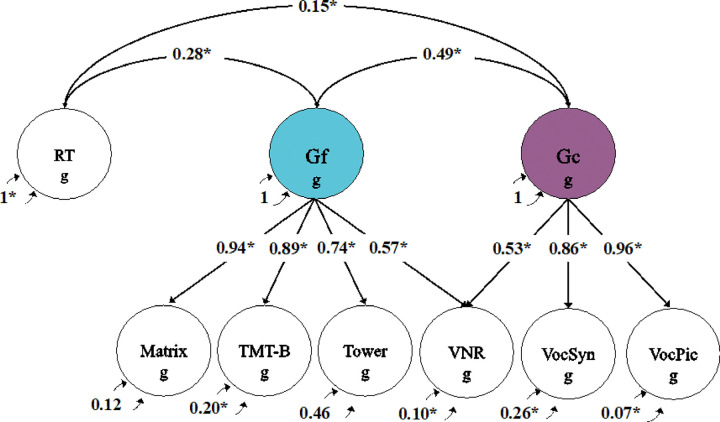
Confirmatory genomic structural equation model of performance on seven cognitive tests. Gf and Gc represent latent variables inferred from the data. RT, reaction time; Gf, fluid intelligence; Gc, crystallized knowledge; Matrix, matrix pattern recognition; TMT-B, trail making test-B; Tower, tower rearranging; VNR, verbal numerical reasoning; VocSyn, synonyms vocabulary; VocPic, picture vocabulary. All indicators are represented as circles, reflecting a genetic component that is not directly measured but inferred from LDSC. The g subscript is used throughout the path diagrams to denote that the variables in these models are strictly defined by the genetic variance captured by current GWAS estimates. Asterisks (*) represent significant estimates (p < 0.05). Standardized parameter estimates are displayed.

We first validated the theoretical expectation of separable RT, Gf, and Gc components of cognitive function by applying a confirmatory genetic factor model to GWAS summary statistics for 7 cognitive tests (all cognitive phenotypes listed in Table 1, excluding the four language phenotypes, which we include subsequently) using Genomic SEM (Fig. 1). Of these 7 cognitive tests, 6 were administered by UK Biobank (UKB), and 1 (synonyms vocabulary) was administered by 23andMe, Inc. (see “Contributing Univariate GWAS: Cognition” in Table 1; Methods for more details). Following well-established empirically-based taxonomies ^16,40–42^, we specified matrix pattern recognition (matrix), tower rearranging (tower), and trail making test-B (TMT-B) as indicators of Gf; and picture vocabulary (VocPic) and synonyms vocabulary (VocSyn) as indicators of Gc. Verbal numerical reasoning (VNR) draws on both reasoning and learned knowledge^43^, and evinces an age trend (increases in performance through midlife) most similar to the established age trend for Gc^22^. It was therefore specified as an indicator of both Gf and Gc. RT was directly indexed by a single measure.

Our confirmatory model closely approximated the observed genetic covariance matrix, as indicated by the fit indices (χ^2^(11) = 49.483, P < 0.0001; CFI = 0.989; SRMR = 0.052). Estimated genetic correlations between the cognitive dimensions were less than 0.5 (RT-Gf = 0.277 [95% CI = 0.214–0.341]; RT-Gc = 0.154 [95% CI = 0.090–0.213]; Gf-Gc = 0.493 [95% CI = 0.403–0.572]), indicating that the cognitive dimensions are genetically distinct.

### Extension of confirmatory factor model to include educational attainment and tests of language

**Figure 2:**
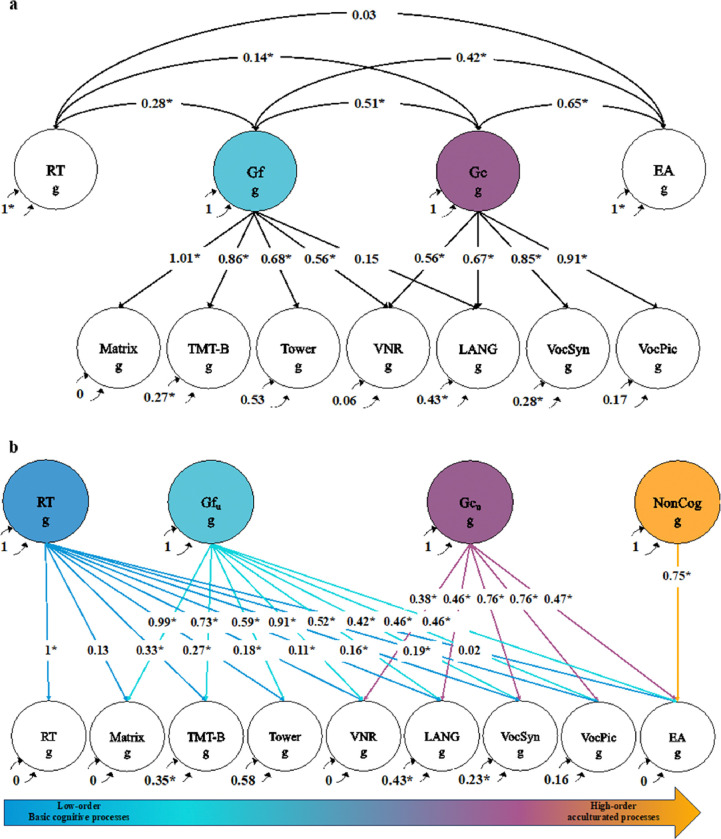
Genomic SEM models for genetic factors related to cognitive and noncognitive skills. a) Extended confirmatory model including educational attainment (EA) and language (LANG). b) Decomposition of genetic variance shared across and unique to cognitive and noncognitive dimensions. The residual variances of RT and EA are fixed to 0, so that all variance is explained by the latent factors. The covariances between latent factors are fixed to 0 in order to find their unique variance in the Cholesky decomposition. All indicators are represented as circles, reflecting a genetic component that is not directly measured but inferred from LDSC. The g subscript is used throughout the path diagrams to denote that the variables in these models are strictly defined by the genetic variance captured by current GWAS estimates. Asterisks (*) represent significant estimates (p < 0.05). RT, reaction time; Gf, fluid reasoning; Gc, crystallized knowledge; Gf_u_ fluid reasoning unique of RT; Gc_u_, crystallized knowledge unique of RT and Gf_u_; NonCog, noncognitive education-related factor; Matrix, matrix pattern recognition; TMT-B, trail making test-B; Tower, tower rearranging; VNR, verbal numerical reasoning; LANG, language factor; VocSyn, synonyms vocabulary; VocPic, picture vocabulary; EA, educational attainment. Standardized parameter estimates are displayed.

Next, we tested whether we could incorporate recent GWASs related to language skills^32^ into our genomic factor model. As a preliminary step, we used GWASs of word reading, nonword reading, phoneme awareness, and spelling to estimate a Language factor (Supplementary Fig. 1). That model fit well (χ^2^(2) = 1.148, P = 0.56; CFI = 1; SRMR = 0.014), with standardized loadings all ≥ .95, supporting the genetic one-dimensionality of performance on these tests. We used this factor model to perform a multivariate GWAS meta-analysis on the language phenotypes within Genomic SEM (Mean χ^2^(1) of Language factor = 1.145). The confirmatory genetic factor model of RT, Gf, and Gc was then expanded to include these GWAS summary statistics for Language and educational attainment, which is known to be genetically and phenotypically related to cognitive function. We expected the language to function as an indicator of Gc, but to ensure that any potential unexpected relation with Gf was accounted for, we also allowed for it to load on Gf. Educational attainment was specified as separate correlated factor (Fig. 2a). The expanded confirmatory factor model closely approximated the observed genetic covariance matrix, as indicated by the fit indices (χ^2^(21) = 92.900, P < 0.0001; CFI = 0.992; SRMR = 0.057). Consistent with expectations, Language had a strong and significant standardized loading on Gc (λ = 0.67, 95% CI = 0.54–0.80) but not Gf (λ = 0.15, 95% CI = −0.01–0.30). Educational attainment exhibited moderate genetic correlations with both Gf (*r* = 0.42, 95% CI = 0.36−0.48) and Gc (*r* = 0.65, 95% CI = 0.61−0.69) but not with RT (*r* = .03, 95% CI =−0.01–0.06).

Next, we re-parameterized the expanded confirmatory factor model as a GWAS-by-subtraction model, in which each factor represents variance shared among the indicators that is unique of (and hence uncorrelated with) the cognitive functions entered earlier in the decomposition (Fig. 2b). We entered more basic cognitive functions earlier than higher-order cognitive functions. Following cognitive theory, we consider RT to be the most basic of the processes, and we consider Gf to be more basic than Gc because the former constitutes abstract reasoning using novel stimuli, whereas the latter constitutes the application of learned concepts to familiar stimuli. We consider educational attainment to be the least basic, due to its social dependence. Following Demange et al.^25^ and previous economic theory ^44^, the unique genetic variance in educational attainment not shared with any cognitive tests was labeled “NonCog”, i.e., non-cognitive skills relevant for education. Note, however, that this NonCog factor differs somewhat from that of Demange et al. ^25^, in that three genetic components of cognitive function (RT, Gf, and Gc), rather than a single composite index of cognitive function, are subtracted from educational attainment. Results indicated that 43.75% of the genetic variance in EA was attributable to the NonCog factor (1-.75^2^), compared to 57% as reported by Demange et al ^25^. This re-parameterized model closely approximated the observed genetic covariance matrix, as indicated by the fit indices (χ^2^(15) = 49.650, P < 0.0001; CFI = 0.996; SRMR = 0.044).

### Identification of novel loci associated with crystallized knowledge (Gc)

Our integration of multiple measures of Gc constitutes the first opportunity to conduct GWAS meta-analysis of Gc. We therefore fit multivariate GWAS within Genomic SEM to identify SNPs (1) associated with Gc (Fig. 2a) and (2) uniquely associated with Gc and not with Gf or RT (i.e., with Gc_u_, Fig. 2b).

Using the method described by Grotzinger et al. ^15^ (section S6), we find that the multivariate GWAS of Gc was powered equivalent to a univariate GWAS with $\hat{N}=438,528$. The mean χ^2^(1) statistic was 1.594. We identified 4,777 genome-wide significant SNPs (P < 5 × 10^−8^), distributed in 78 independent genome-wide significant loci. Eight (10%) of those loci were not previously associated with any of the eight cognitive phenotypes that served as the basis for the genomic SEM model, and 5 of them were novel to any cognitive trait in the GWAS catalog. One of them was in an intergenic region on chromosome 1, near gene RP11–459K23.2 with no phenotypic associations in the GWAS catalog. Another one was in an intergenic region on chromosome 2, near genes *C2orf73* and *SPTBN1*. The identified SNPs have been associated with granulocyte telomere length, memory T-cell telomere length and bone mineral density in spine. An additional set of SNPs are in an intergenic region on chromosome 11, between genes *RPL12P46* and *LINC02698*, previously only associated with heel bone mineral density. The pleiotropic associations of common variants with both cognitive function and bone density parallel results from recent studies of rare variants: A study of rare protein coding variation in relation to verbal-numerical reasoning in the UKB also identified a genetic locus (*KDM5B*) with pleiotropic effects on bone mineral density, and *KDM5B* mutant mice showed both cognitive deficits and skeletal abnormalities^45^.

SNPs in another locus on chromosome 11, near genes GRM5 and TYR, have been associated with skin cancer, melanoma, skin sensitivity to sun, insomnia, tan response, sunburns and hearing loss. This association might reflect uncontrolled population stratification and/or observable physical traits linked to discrimination in schooling. Finally, SNPs in the genetic region of chromosome 14 near genes *PPP1R13B, ZFYVE21* and *LINC00637* have been previously associated with smoking, risk taking, problematic alcohol use, number of sexual partners, sex hormone-binding levels, BIP, SZC and insomnia.

Even though the multivariate GWAS on Gc is based on a model where Gc are allowed to covary with Gf, the two dimensions of cognitive function shared only one hit, a locus on chromosome 3, a long region of 1,2 Mb encompassing 58 genes. This locus was also shared with NonCog, VNR, VocSyn, EA4 and ADHD, and the SNPs in this region have been associated with multiple metabolic and cognitive traits. This locus was not associated with unique variance in Gc (Gc_u_, see next section), which suggests that the tagged SNPs in this region may only be related to Gc through their effects on Gf, what Cattell termed fluid investment^46^.

### Identification of novel loci associated with crystallized knowledge unique of reaction time and fluid reasoning (Gc_u_)

The multivariate GWAS of Gc(Gc_u_) unique of RT and Gf was powered equivalently to a univariate GWAS with $\hat{N}=172,623$. The mean χ^2^(1) statistic was 1.467 We found 289 genome-wide significant SNPs (P < 5 × 10^−8^), distributed in 9 independent genome-wide significant loci. Two of those loci were not previously associated with any of the eight indicators of cognitive abilities that went into the Genomic SEM model, nor any cognitive trait in the GWAS catalog. The candidate SNP *rs35225412* and *rs35225412*, mapped to the intergenic region between the genes *C2orf73* and *SPTBN1* on chromosome 2, have not associated with any trait previously, whereas the SNPs found in the intergenic region 7:114337615–114428727, near the *FOXP2* gene, have been associated previously with body mass index, Chron’s disease, pelvic organ prolapse and general risk tolerance.

We observed a few loci that exhibit patterns of association inconsistent with our genome-wide model. Specifically, there were cases where a locus was associated with both a cognitive factor and NonCog, despite NonCog being specified to be genetically orthogonal to the cognitive factors. Although these cases are rare, they may reflect separate genetic effects among correlated linked loci, or loci that exert effects on EA beyond what is expected through cognitive pathways alone. For instance, locus 6 of NonCog is associated with locus8 VocSyn, and locus 75 EA, suggesting an association between VocSyn and EA not mediated by cognitive pathways.

The overlap between genetic loci associated with multivariate factors in the current analysis (e.g., Gc, Gc_u_) and those discovered in previous univariate GWAS (e.g., with educational attainment) is summarized in Supplementary Tables 3 and 4, and is reported in more detail in Supplementary Tables 6 to 21. In summary, the multivariate GWAS approach identified novel genetic loci (8 for Gc; 2 for Gc_u_) not previously discovered in univariate GWASs of individual cognitive abilities and distinct across different dimensions of cognitive ability.

### Genetic associations between cognitive factors and neuropsychiatric disorders

We extended the model shown in Fig. 2b (see Supplementary Fig. 2) to estimate genetic associations between each of the cognitive/noncognitive factors (RT, Gf_u_, Gc_u_, and NonCog) with seven external variables: five neuropsychiatric disorders (SCZ, BIP, AUT, ADHD, and ALZ) and two personality dimensions relevant to education (Conscientiousness and Openness).

**Figure 4:**
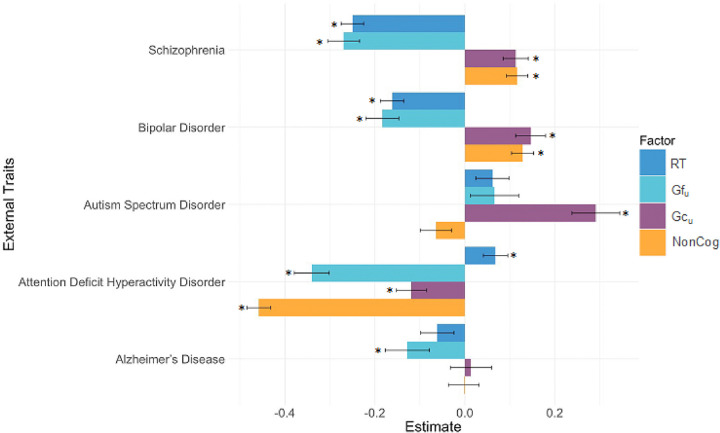
Genetic correlations of neuropsychiatric disorders with cognitive and noncognitive factors. Asterisks represent significant genetic correlations between the neuropsychiatric disorder and the factor. Error bars represent standard errors.

The disorders exhibited highly heterogeneous patterns of genetic associations with cognitive abilities and the noncognitive factor, such that genetic correlations differed in both magnitude and direction across disorders (Fig. 4, Supplementary Table 5). Schizophrenia and bipolar disorder displayed the most similar patterns of association: Both showed negative genetic correlations with RT and Gf_u_ (rGs ranging from −0.16 to −0.28), but positive associations with Gc_u_ and NonCog (rGs ranging from 0.11 to 0.15). In contrast, ADHD exhibited a slight positive association with RT, but negative associations with Gf_u_ Gc_u_, and NonCog (rGs ranging from − 0.07 to −0.46). Autism Spectrum Disorder displayed positive associations with Gc_u_. Alzheimer’s Disease was only associated with Gf_u_.

Some of these genome-wide heterogeneous associations can also be seen when we zoom in at the genome-wide independent loci. For instance, in all Gf significant independent loci overlapping with SCZ significant independent loci (loci 5,7,11 for Gf and 41, 71, 100 for SCZ; Supplementary Table 14) risk alleles for schizophrenia are associated with deficits in fluid cognition. On the other hand, locus 66 of Gc defined by the lead SNP rs12433109 overlaps with locus 145 in SCZ (Supplementary Table 15). The A allele of this SNP has an Z score of 6.72 for SCZ and 4.23 for Gc, meaning that even though it increases the risk for schizophrenia it is also associated with enhanced crystallized knowledge.

These findings suggest complex and diverse genetic relationships between neuropsychiatric disorders, and cognitive and noncognitive factors associated with EA. In particular, we find multiple instances of differently signed associations between a given neuropsychiatric disorder and different cognitive and noncognitive factors, and the patterns vary considerably across disorder.

Finally, regarding personality traits, Openness to Experience (which taps intellectual curiosity ^47^ and engagement with learning opportunities) was moderately associated with Gc_u_ (*r*_*g*_ = 0.47, 95% CI = 0.38/0.55) and weakly associated with lower Gf_u_ (*r*_*g*_ = 0.03, 95% CI =−0.17/−0.01) and RT (*r*_*g*_ = 0.092, 95% CI = 0.02/0.16). Conscientiousness (which taps organization, planning and self-regulation ^48^) was positive associated with NonCog (*r* = 0.21, 95% CI = 0.15/0.26) and weakly associated with lower Gf_u_ (*r* = −0.04, 95% CI =−0.18/−0.03).

## Discussion

Cognitive function has often been treated as a single entity in genetic research^5,6,45,49^, which obscures the differential impacts that specific cognitive domains may have on neuropsychiatric conditions. Evidence from research outside of genomics consistently suggests that cognitive impairments in neuropsychiatric disorders are best characterized at the level of cognitive domains, rather than as uniform deficits in a single general ability. However, this differentiation has not been directly tested at the domain level in multivariate genome-wide studies, making it challenging to parse out unique patterns of association, especially with respect to the fluid versus crystallized components. This study represents the first such multidimensional analysis of cognitive function in the genetic context. We performed a multivariate GWAS on the crystallized knowledge domain (Gc) and the unique variance associated with crystallized knowledge (Gc_u_) when processing speed (measured by tests of reaction time, RT), and fluid reasoning (Gf) are accounted for. To our knowledge this is the first multivariate GWAS on Gc and Gc_u_. We went on to use this multidimensional approach to characterize the specific and heterogeneous relationships between neuropsychiatric disorders and cognitive ability and education-related noncognitive skills.

### Novel insights into genomic loci associated with crystallized knowledge

Our GWAS of crystallized knowledge (Gc) identified 78 significant loci, including five that are novel to any cognitive trait. The discovery of loci associated with cognitive function, biobehavioral phenotypes and traits like bone mineral density suggests potential pleiotropic effects. For example, shared genetic loci between Gc and bone mineral density might indicate a link to structural brain properties or shared developmental pathways. This is supported by experimental studies^45^ showing that mice with abnormal expression of KDM5B, a genetic locus linked to verbal-numerical reasoning (VNR), exhibit both cognitive deficits and skeletal abnormalities. Future studies can use a similar approach to generate mutants for the novel loci we detected for Gc to assess their pleiotropic effects.

### Genetic Associations with Neuropsychiatric Disorders

Our findings highlight the importance of considering cognitive function at the domain level, as we identify heterogeneous and disorder-specific genetic associations with different cognitive dimensions, which vary both in magnitude and direction across disorders. For instance, genetic risk for Schizophrenia and bipolar disorder was associated with deficits in RT and Gf_u_, but enhanced Gc_u_ and NonCog. This observation is consistent both with the fluid deficits often associated with psychotic disorders, and with the common observation that relatives of individuals with such disorders commonly display enhanced creativity, curiosity, and achievement. In contrast, genetic risk for ADHD was associated with enhanced RT, possibly reflecting less inhibited responding, but deficits in Gf_u_, Gc_u_, and NonCog. Autism Spectrum Disorder displayed positive associations with Gc_u_ but negative associations with NonCog. With respect to Autism Spectrum Disorder, we further refine the established positive genetic association with cognitive function^36^, finding it to be specific to crystallized knowledge. De novo mutations may play an especially strong role for AUT, and AUT is clinically associated with impairments in cognitive function and language. It is therefore possible that this paradoxical positive genetic correlation between AUT and crystallized knowledge does not generalize to the rare variant spectrum. Finally, Alzheimer’s Disease was only associated with Gf_u_, which may reflect both the fact that fluid reasoning tests (so-called executive tests) are often more sensitive than tests of Gc and RT to dementia, and the fact that neuropsychological instruments tapping fluid abilities may be more strongly relied upon in clinical diagnosis of dementia. Together, these findings challenge prior conclusions drawn from aggregated cognitive measures and emphasize the need for more targeted approaches to understand the relationship between cognition and psychopathology.

The heterogeneous associations between cognitive factors and neuropsychiatric disorders may also relate to the evolutionary paradox where common genetic variants linked to neuropsychiatric disorders have survived negative selection. If risk alleles for a psychiatric disorder confer a positive impact on one domain cognitive function, or noncognitive skills for educational attainment (EA), but yield neutral or negative effects on another, balancing selection mechanisms may maintain these alleles at moderate frequencies within human populations ^50^. These positive associations could also be significant for applications of polygenic scores, as selecting against SCZ, BIP, or ADHD genes might unintentionally reduce cognitive function in at least one dimension. For instance, in rs 12433109 selecting against the A risk allele for schizophrenia could result also in selecting for deficits in crystallized knowledge. Furthermore, these results may have implications for drug development and repurposing, recent findings suggest that drugs supported by positive GWAS are more likely to succeed in trials ^51^, but trials for neuropsychiatric drugs might also need to consider unexpected impacts on particular dimensions of cognition.

### Genetic Associations with personality traits

In line with recent findings on the genetic architecture of personality traits, our results underscore the importance of domain-specific genetic associations when exploring the relationship between cognitive functions and neuropsychiatric disorders. We found that Openness, a trait linked to intellectual curiosity and creativity, exhibited strong genetic associations with crystallized knowledge (Gc), and minimal associations with fluid ability (Gf) and reaction time (RT). This observation is consistent with theories of knowledge acquisition, whereby individuals who are intrinsically curious and motivated to explore and learn from their environments seek out and attend to opportunities to acquire such knowledge ^52,53^. Shared genetic architecture between crystallized knowledge and neuropsychiatric disorders, such as SCZ and BP, may reflect the strong connection known to exist between these disorders and curiosity and openness to experience in the general population ^26,54^.

We also found that Conscientiousness, defined by traits such as organization, persistence, and self-regulation, was genetically associated with non-cognitive factors that influence educational attainment. Gupta et al.^33^ found two genome-wide significant loci associated with Conscientiousness, one of which involves the *FOXP2* gene, a gene with well-established associations with language^55^. Here, we find that FOXP2 also linked to EA, Gc, Gc_u_, and NonCog. The persistence of *FOXP2*s association with NonCog, even after controlling for the influence of Gc or Gc_u_ on EA, suggests that while some of *FOXP2*s effects on EA may be mediated by crystallized knowledge, its role extends beyond Gc.

### Limitations and Conclusions

This study has several limitations. First, the GWAS primarily involved individuals of European ancestry, which may limit the generalizability of the findings to other populations. Additionally, the results might still be confounded by uncorrected population stratification, especially given that one of the hits for Gc was related to skin tone. Finally, the potential impact of assortative mating on genetic correlations is uncertain and requires further investigation.

Moreover, while we report overlap between loci associated with cognitive domains, it is crucial to note that physical proximity of loci does not necessarily imply functional overlap. The clumping algorithm we used, based on either loci within 250 Kb or low recombination rates (r < 0.1), may identify loci that are physically close but not functionally related. This could result from non-functional physical proximity or phenomena like assortative mating in the reference populations used in the clumping algorithm of FUMA. Therefore, interpreting these overlaps functionally should be approached with caution.

In conclusion, our multidimensional approach to cognitive function and its genetic underpinnings provides nuanced insights into the shared genetic architecture between cognitive abilities and neuropsychiatric disorders. By performing a multivariate GWAS of crystallized knowledge (Gc) alongside fluid ability and reaction time, we underscore that cognitive ability is not a uniform entity.

## Methods

### GWAS summary statistics

We used GWAS summary statistics for cognitive abilities, educational attainment, and neuropsychiatric disorders to fit a Structural Equation Model (SEM) using the Genomic SEM package in R v.4.1.1^31^. Sample size, Mean χ^2^, LDSC intercept and SNP heritability of each summary statistic are reported in Table 1. All the GWAS summary statistics used in this study come from individuals of European (EUR) Ancestry.

#### *Cognitive abilities*.

We selected the GWAS summary statistics of six cognitive tests from UKB, five of them curated by de la Fuente (2021)^14^ as indicators for our SEM model:

1. *Reaction time* ^14^ was a self-administered test completed by participants at the baseline UKB assessment. In this task, pairs of either identical or different cards were presented on a computer screen. If the two cards were identical, participants had to push a button as quickly as possible. Reaction time score corresponded with the time, in milliseconds, to identification of matching cards in four trials. Participants were presented with 12 trials in total. The first five trials were used as a practice. Of the remaining seven trials, four presented identical cards. The score is the mean time (in milliseconds) for these four trials. Whereas there were only a few trials, internal consistency is good (Cronbach α = 0.85). Scores were multiplied by − 1 such that higher scores indicated more optimal performance.
2. *Matrix pattern recognition* ^14^ was assessed using the non-verbal fluid reasoning matrix pattern recognition test, an adaptation of the matrices test included in the COGNITO battery^56^, which is similar to the well-known Raven’s progressive matrices test. This test was self-administered during the assessment center imaging visit. This test involves the inspection of an abstract grid pattern with a piece missing in the lower right-hand corner. The pattern has a logical order. The participant is asked to select the correct multiple-choice option at the bottom of the screen to complete the logical pattern both horizontally and vertically. This 15 -item test aims at assessing the ability to solve non-verbal, non-numerical problems using novel and abstract materials. The score is the total number of items solved correctly in 3 min.
3. *Tower rearranging* ^14^ was assessed with a self-administered test during the imaging assessment center visit. It is like the well-known ‘tower of Hanoi’ task. Participants were presented with a display (display A) containing three different colored hoops arranged on three pegs (towers). Another display (display B) was shown underneath display A, with the three hoops arranged differently. The task involves deciding how many moves it would take to change display A into display B. The score was the number of correctly completed trials achieved in 3 min.
4. *Trail making test*-B^14^ is a computerized version of the Halstead-Reitan trail-making test^57^. The trail-making test was self-administered during both the assessment center imaging visit and the web-based cognitive assessment. In part B of the test, participants were presented with the numbers 1–13 and the letters A-L arranged quasi-randomly on a computer screen. The participants were instructed to switch between touching the numbers in ascending order, and the letters in alphabetical order (for example, 1-A-2-B-3-C) as quickly as possible. The score was the time (in seconds) taken to successfully complete the test. Those with a score coded as 0 (denoting trail not completed) had their score set to missing. Scores were multiplied by − 1 such that higher scores indicated more optimal performance. In this analysis, the scores used were from the first testing occasion for each participant.
5. *Verbal numerical reasoning*^14^ was self-assessed at the baseline assessment center visit by a subsample of UKB participants. Participants were asked 13 multiple-choice questions that assessed verbal and numerical problem solving. The score was the number of questions answered correctly in 2 min. This test has been shown to have adequate test–retest reliability (r = 0.65)^58^ and internal consistency (Cronbach α = 0.62)^22^. The verbal numerical reasoning test was also administered to three subsamples of participants at the first repeat assessment visit, the assessment center imaging visit and during the web-based cognitive assessment. In the web-based version of this test because there was an additional question the maximum score was 14. In the current analysis the verbal numerical reasoning score used is taken from the first testing occasion for each participant.
6. *Picture vocabulary* (in-house GWAS), was assessed the UK Biobank using an adaptation of the NIH Toolbox Picture Vocabulary Test ^59^, originally designed for a US audience. This test correlated very highly (r = 0.83) with the original version of this test^43^. In the UK Biobank version, participants are shown a sequence of screens with words and four corresponding pictures on a touchscreen interface. Unlike the NIH version, there is no audio component in the UK Biobank test; participants only see the visual depiction of the words. Data collected during the test include the word presented at each round, the participant’s selected picture, response time, final vocabulary level estimate, and standard error of the estimate. The difficulty levels of words were calibrated initially for a US audience but were later recalibrated for the UK Biobank context, resulting in additional derived data related to cognitive function and visuomotor processing times. The UK Biobank version retained the dataset of words and pictures from the NIH version but made some modifications to adapt to UK English, such as altering words to match UK counterparts and removing ambiguous or culturally specific words. The testing process also differs slightly from the US version, with adjustments in the start and end conditions of the test
7. *synonyms vocabulary*^23^, assesses verbal reasoning and long-term verbal memory by requiring participants to identify word synonyms. Modeled after the Wordsum task used in General Social Surveys ^60^, participants select the word most similar in meaning to a target word from a list of five options. The primary outcome is the number of correct answers out of 20 questions. This test provides a measure of crystallized cognitive function, reflecting accumulated word knowledge across one’s lifespan and being less influenced by short-term health changes. Internal split-half reliability in the sample was adequate (r = 0.69) ^23^.

#### Language abilities.

Language abilities were measured using **1)***word reading*: number of correct words read aloud from a list in a time-restricted or unrestricted fashion; **2)***nonword reading*: number of nonwords (group of phonemes that looks or sounds like a word, obeys the phonotactic rules of the language, but has no meaning) read aloud correctly from a list in a time-restricted or unrestricted fashion; **3)***spelling*: number of words correctly spelled orally or in writing after being dictated as single words or in a sentence; and **4)***phoneme awareness*: number of words correctly altered in phoneme deletion/elision and spoonerism tasks ^32^.

#### Educational attainment.

We used the summary statistics from the meta-analysis, made by Okbay et al. ^39^ which resulted from combining data from UKB, 23andMe and other 69 cohorts. The phenotype was measured as number of years of schooling completed.

#### Neuropsychiatric disorders.

We obtained most of the data from the Psychiatric Genetics Consortium (PGC) database ^61^. To calculate an unbiased estimate of liability scale heritability, we used the effective population size (Neff) calculated based on the approach described in Grotzinger et al. ^15^. Neff represents the sample size of a GWAS with 50% of cases and 50% of controls and is calculated as ${Neff}_{k}=4V_{k}(1-Vk)n_{k}$, where k is the GWAS, $V_{k}$ is the ratio of cases of each cohort, and $n_{k}$ is the cohort total sample size. The Neuropsychiatric disorders GWAS summary statistics included were Schizophrenia ^34^, Bipolar disorder ^35^, Autism spectrum disorder ^36^, Attention deficit hyperactivity disorder ^37^, Alzheimer’s disease ^38^.

#### Personality traits.

The genetic and phenotypic data for these phenotypes was taken from Million Veteran Program (MVP) ^62^ release version 4 as described in Gupta et al. (2024) ^33^. A 10-item scale measuring the Big Five personality traits (BFI-10) was included as part of a self-report Lifestyle survey provided to MVP participants, with two items for each of the personality traits. We only used data from individuals of EUR ancestry whose mean age was ~ 65.5 years for each of the traits and 8% of the sample was female.

### Genomic SEM

We used Genomic SEM v.0.0.5^31^ in R v.4.3.2 to fit Structural Equation Models (SEMs) using the GWAS summary statistics mentioned above. Table 1 reports the input GWAS information, population size, mean χ^2^, LDSC intercept, and estimated SNP heritability. Genomic SEM is a two-step SEM approach. First, we use multivariate linkage disequilibrium score regression (LDSC) ^63^ to estimate the genetic covariance matrix (S) for the phenotypes along with the associated matrix of sampling variances and covariances (V). We used the ‘munge’ function in Genomic SEM on the summary statistics to convert them to the format expected by LDSC, using the HapMap3 (~ 1.2 million SNPs) as reference. In the second step, an SEM is fit to the S matrix using weights determined from the V matrix. We used unit variance identification for all models where factors were involved except in the case where SNP effects were added to the models to perform a multivariate GWAS. Genetic correlations with external traits (modeled in Supplementary Fig. 2, results in Fig. 4 and Supplementary Table 5) were estimated in separate models, having only one external trait in each model, instead of estimating all the correlations at once.

### Multivariate GWASs

For every GWAS summary statistic used in a model with SNP effects, we examined the phenotypic standard errors to ensure the expected relationship between standard errors and sample size, assuming phenotypes were standardized. For any deviations, effect sizes (BETA) and standard errors (SE) were rescaled. Specifically, minor allele frequencies (MAF) were used to calculate the variance of the SNP (varx) under Hardy-Weinberg equilibrium. The variance of the phenotype (sdy) was then derived from SE, BETA, MAF, and the sample size (N). If the median sdy was not approximately 1, indicating non-standardization, the BETA and SE were divided by the median sdy to standardize the phenotypic variance.

We added SNP effects to the model depicted in Fig. 2 and Supplementary Fig. 1, using unit loading identification to freely estimate the variance of the factors and estimate the variance explained by each SNP. For the Language GWAS we specified the unstandardized loading of nonword reading to be 1 (Supplementary Fig. 1); for the correlated model of Fig. 2a, we dropped the path form Gf to Language, and the indicators with specified unstandardized loadings of 1 were Matrix (for Gf) and VocSyn (for Gc; whereas for re-parameterized model of Fig. 2b, they were RT (for RT), Matrix (for Gf), VocSyn (for Gc) and EA (for NonCog).

### FUMA analyses

We used FUMA v1.5.2^64^ SNP2GENE function to identify independent genome-wide-significant loci. SNPs are considered as independent hits or independent genome-wide-significant loci if they are within a 250 kb window and linked (r2 < 0.1). FUMA also considers all the SNPs that are linked to the independent hits (r > 0.6), irrespective of being significant or not in the input GWAS (we set this argument manually, P-value cutoff = 1) to label them as candidate SNPs and identify related genes and traits in the GWAS catalog. We also use this list of candidate SNPs to detect overlaps between independent hits. This way, a factor does not share independent hits with an indicator if the independent hits from both are not shared, but also if their linked SNPs (r > 0.6, irrespective of their P value) are not shared.

## Figures and Tables

**Figure 3:**
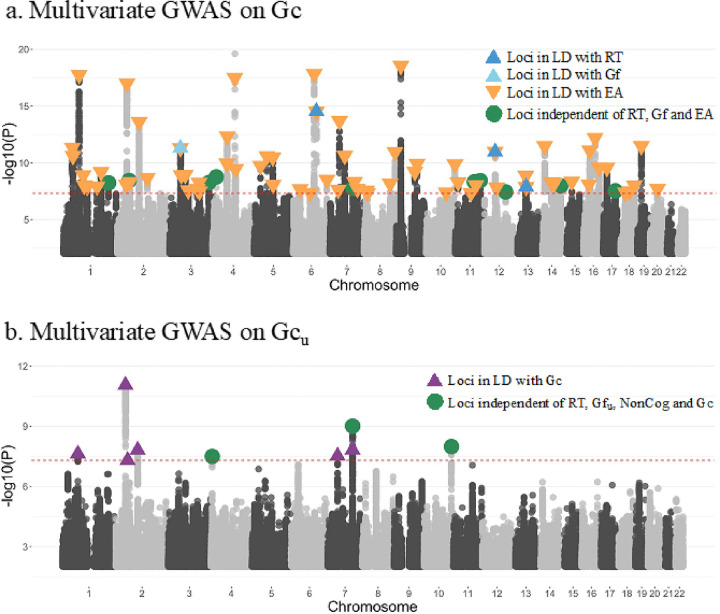
Manhattan plots for Gc. a) Genome-wide significant loci associated with Gc (78 total) as modeled in Figure 2a; blue, light blue and orange triangles represent that are in LD with RT (3), Gf (1) and EA (68) respectively. Green dots represent the genome-wide significant loci that are not in LD with RT, Gf or EA (10 independent loci). b) Genome-wide significant loci uniquely associated with Gc (Gc_u_, 9) as modeled in Figure 2b. Purple triangles represent genome-wide significant loci in LD with Gc (6) as modeled in Figure 2a. Green dots represent the genome-wide significant loci that are not in LD with Gc (3 independent loci). Red line represents the genome-wide significance threshold (P < 5× 10^−8^). Loci are considered in LD if their LD was r2 > 0.10 or within a 250-kb window of one another, otherwise they are considered independent loci.

**Table 1:** GWAS summary statistics related to cognition, education and neuropsychiatric disease

Phenotype	Samples Size	Mean χ^2^(1)	LDSC intercept	SNP heritability	Significant independent loci
Cognitive Phenotypes	N				
Reaction time (Processing Speed) ^14^	330,024	1.420	1.021	0.074	39
Matrix pattern recognition (Gf) ^14^	11,356	1.040	1.013	0.147	0
Tower rearranging (Gf) ^14^	11,263	1.022	1.008	0.120	0
Trail making test-B (Gf) ^14^	78,547	1.203	1.000	0.153	7
Verbal numerical reasoning (Gf and Gc) ^14^	171,304	1.639	1.021	0.217	89
Vocabulary synonyms (Gc) ^23^	188,434	1.246	1.016	0.103	25
Vocabulary pictures (Gc) ^in-house GWAS^	28,337	1.031	1.009	0.208	3
Word reading (Language/Gc) ^32^	33,959	1.080	1.000	0.210	0
Nonword reading (Language/Gc) ^32^	17,984	1.065	0.994	0.273	0
Phoneme awareness (Language/Gc) ^32^	13,633	1.035	0.998	0.250	0
Spelling (Language/Gc) ^32^	18,514	1.063	0.995	0.242	0
Non-Cognitive Phenotypes	N				
Educational attainment ^39^	3,037,499	5.473	1.636	0.104	1549
Openness to experience ^33^	220,015	1.206	0.993	0.048	7
Conscientiousness ^33^	234,880	1.248	1.020	0.048	2
Neuropsychiatric Disorders	N cases/controls (Sum of Effective N)				
Schizophrenia ^34^	53,386/77,258 (117,494)	1.910	1.072	0.225	179
Bipolar disorder ^35^	41,917/371,549 (101,963)	1.500	1.033	0.183	60
Autism spectrum disorder ^36^	18381/27,969 (43,778)	1.162	1.009	0.117	3
Attention deficit hyperactivity disorder ^37^	38,691/186,843 (103,135)	1.401	1.027	0.203	27
Alzheimer’s disease ^38^	(76,510)	1.139	0.994	0.073	24

* Sum of effective N comes from De la Fuente et al. (2021) ^38^ in which SNP heritability and SNP effectswere appropriately estimated and produced consistent results when combining GWAS and GWAXsummary data.
